# Disparities in routine healthcare utilization disruptions during COVID-19 pandemic among veterans with type 2 diabetes

**DOI:** 10.1186/s12913-023-09057-8

**Published:** 2023-01-16

**Authors:** Samrachana Adhikari, Andrea R. Titus, Aaron Baum, Priscilla Lopez, Rania Kanchi, Stephanie L. Orstad, Brian Elbel, David C. Lee, Lorna E. Thorpe, Mark D. Schwartz

**Affiliations:** 1 Department of Population Health, New York University Grossman School of Medicine, 180 Madison Avenue, 4th Floor, #4-54, New York, NY 10016 USA; 2 Department of Global Health, Icahn School of Medicine at Mount Sinai, New York, NY USA; 3 Department of Medicine, New York University Grossman School of Medicine, New York, NY USA; 4 Wagner Graduate School of Public Service, New York University, New York, NY USA; 5 Ronald O. Perelman Department of Emergency Medicine, NYU Grossman School of Medicine, New York, NY USA; 6 VA New York Harbor Healthcare System, New York, NY USA

**Keywords:** Health disparity, Covid-19 pandemic, Type 2 diabetes care, Veterans, Telehealth visits

## Abstract

**Background:**

While emerging studies suggest that the COVID-19 pandemic caused disruptions in routine healthcare utilization, the full impact of the pandemic on healthcare utilization among diverse group of patients with type 2 diabetes is unclear. The purpose of this study is to examine trends in healthcare utilization, including in-person and telehealth visits, among U.S. veterans with type 2 diabetes before, during and after the onset of the COVID-19 pandemic, by demographics, pre-pandemic glycemic control, and geographic region.

**Methods:**

We longitudinally examined healthcare utilization in a large national cohort of veterans with new diabetes diagnoses between January 1, 2008 and December 31, 2018. The analytic sample was 733,006 veterans with recently-diagnosed diabetes, at least 1 encounter with veterans administration between March 2018–2020, and followed through March 2021. Monthly rates of glycohemoglobin (HbA1c) measurements, in-person and telehealth outpatient visits, and prescription fills for diabetes and hypertension medications were compared before and after March 2020 using interrupted time-series design. Log-linear regression model was used for statistical analysis. Secular trends were modeled with penalized cubic splines.

**Results:**

In the initial 3 months after the pandemic onset, we observed large reductions in monthly rates of HbA1c measurements, from 130 (95%CI,110–140) to 50 (95%CI,30–80) per 1000 veterans, and in-person outpatient visits, from 1830 (95%CI,1640–2040) to 810 (95%CI,710–930) per 1000 veterans. However, monthly rates of telehealth visits doubled between March 2020–2021 from 330 (95%CI,310–350) to 770 (95%CI,720–820) per 1000 veterans. This pattern of increases in telehealth utilization varied by community type, with lowest increase in rural areas, and by race/ethnicity, with highest increase among non-hispanic Black veterans. Combined in-person and telehealth outpatient visits rebounded to pre-pandemic levels after 3 months. Despite notable changes in HbA1c measurements and visits during that initial window, we observed no changes in prescription fills rates.

**Conclusions:**

Healthcare utilization among veterans with diabetes was substantially disrupted at the onset of the pandemic, but rebounded after 3 months. There was disparity in uptake of telehealth visits by geography and race/ethnicity.

**Supplementary Information:**

The online version contains supplementary material available at 10.1186/s12913-023-09057-8.

## Background

Emerging studies suggest that the COVID-19 pandemic caused significant disruption in routine healthcare, including reduced in-person primary care visits [1–5]. Gaps in routine care can have a negative impact on health outcomes, especially among those with chronic diseases, such as type 2 diabetes (referred to as diabetes hereafter) [6]. While over 30 million Americans [7], including many veterans [8], are living with diabetes, changes in their care utilization as a result of the pandemic have not been well characterized, particularly with respect to variations by patient characteristics, disease severity, or geography [9]. Understanding the pandemic’s impact on healthcare utilization among patients with diabetes is needed to design strategies to reduce the negative impact that gaps in healthcare services may have on this large, vulnerable population.

While frequent routine care is crucial for improving diabetes control and reducing related complications risks [10, 11], there were challenges providing in-person diabetes management care during COVID-19 [12]. Benefits of routine in-person care include face-to-face patient-provider interactions, optimized setting for lab measurements, and lower technology burden on patients [13]. However when these barriers exist, telehealth may provide alternative solution to replicate in-person care [12, 13]. Many published trials and meta-analyses have shown improved glycemic control and increased frequency of care via telehealth visits among patients with diabetes [14–16]. While telehealth can provide a stopgap solution to loss of in-person services during the pandemic, little is known about which patient subgroups used or benefited from this healthcare alternative.

In March 2020, in response to the initial wave of the pandemic, the Veterans Administration (VA) ordered an almost-complete, national shutdown of in-person outpatient care at VA hospitals and clinics. Shortly thereafter, the VA expanded telehealth care via telephone and video conferencing. While studies have begun to characterize the uptake of telehealth visits [2, 17], the extent to which these disruptions and changes impacted routine care among veterans with diabetes, including regular glycohemoglobin (HbA1C) testing and medication prescription fills, remains unknown.

To address this gap and to inform mitigation strategies, we examined longitudinal trends in routine healthcare utilization during the first year of the pandemic among veterans with diabetes from an existing large national cohort using VA electronic health records (EHR). Using an interrupted time series design with the pandemic onset as an external shock, we quantified the pandemic’s disruption on routine medical care among veterans with diabetes. To determine if baseline levels of glycemic control were associated with subsequent utilization patterns during the pandemic, we compared utilization trends among patients with different baseline glycemia levels. To assess the potential differential impacts of the pandemic on healthcare utilization by patient demographics or geographic context, we compared trends among racial/ethnic groups and across the urban-rural continuum.

## Methods

### Design and data sources

We conducted an interrupted time series analysis using data from the Veteran Affairs’ Corporate Data Warehouse, a national repository of clinical and administrative data, available through the VA Informatics and Computing Infrastructure. We used data from the VA Diabetes Risk (VADR) cohort; a large, established national cohort of 6,082,246 veterans seen for at least two primary care visits in any VA Medical Centers or VA Community Based Outpatient Clinics prior to January 2008, and with at least two additional visits between January 2008 and December 2016, and free of diabetes at cohort entry. The VADR cohort has been described in detail elsewhere [18]. Briefly, the cohort was created as a part of the Diabetes Location, Environmental Attributes, and Disparities (Diabetes LEAD) network, a collaboration between multiple academic institutions aiming to study the role of community level factors on diabetes incidence, funded by the Centers for Disease Control and Prevention (CDC) [19].

We longitudinally examined healthcare service utilization outcomes among veterans who developed a new diagnosis of diabetes during the VADR follow-up time period of January 1, 2008 through December 31, 2018. We defined incident diabetes as any of three criteria: (1) at least two encounters (inpatient or outpatient) with documentation of a diabetes ICD-9/10 code; or (2) a prescription for a diabetes medication other than metformin or acarbose alone; or (3) at least one encounter with a diabetes ICD-9/10 code and two elevated (≥6.5%) HbA1C test results. For the current analysis, in addition to these criteria, we required that the veterans in the analytic cohort have at least one encounter with the VA health system since March 1, 2018 to ensure adequate follow-up. As of December 31, 2018, 936,627 (15.6%) veterans were newly diagnosed with diabetes during a median cohort follow-up of 5.5 years. Of this population, the analytic sample for this study was 733,006 veterans with incident diabetes and at least 1 VA encounter between March 2018 and March 2020. The analytic sample was followed until March 2021 to assess the study outcomes.

### Study variables

*Exposure.* We defined the COVID-19 pandemic onset as March 2020, when stay-at-home orders were issued nationwide [20], and compared outcomes before and after this month. As a secondary analysis, we examined the patterns of utilization across four phases in the first year of the pandemic; March 2018 through February 2020; March through June 2020; July through December 2020; and January through March 2021 [21].

*Utilization Outcomes.* We identified HbA1C test for all individuals in the cohort based on laboratory codes and laboratory testing data and determined monthly counts of HbA1C tests. HbA1C values outside a plausible range (< 3.1 or > 19.5) were excluded, as were observations in which an individual had multiple HbA1C tests recorded in a single day with values that were more than one point apart [22]. We assessed changes in routine care by capturing monthly rates of HbA1C tests performed per 1000 veterans in the analytic cohort. Denominators for computing monthly rates were fixed at the total number of veterans in the analytic cohort or within each subgroup for the subgroup analyses.

We extracted monthly counts of all outpatient visits to any VA facility and categorized these visits as in-person or telehealth (telephone or video) according to decision support identifiers (primary and secondary stop codes; Table S1). In a secondary analysis, we limited these stop codes to identify primary care visits only. Changes in visits were assessed by capturing monthly in-person and telehealth visits per 1000 veterans.

Finally, we extracted monthly counts of prescriptions that were filled or refilled in VA pharmacies for diabetes and hypertension medications based on annual VA national formulary [23] (Table S2). We calculated monthly rates of prescription fills per 1000 veterans separately for diabetes and hypertension medications. For computing monthly prescription fill rates, the denominator included veterans who filled their prescriptions, for each medications separately, in the VA pharmacy at least once between March 2018–2021.

#### Other measures

*Pre-pandemic glycemia:* Veterans were stratified based on their most recent HbA1c measurements prior to March 2020 to characterize their pre-pandemic glycemia in four strata: HbA1c < 5.7, 5.7–6.49, 6.5–8.9, and > 9.0 [24].

*Community type:* We defined four community types, measured at the census tract-level, using strata developed by the authors and others in the LEAD Network, described elsewhere [18]. Briefly, these community types are based on a modification of the Rural-Urban Commuting Area (RUCA) codes from the US Department of Agriculture. After collapsing the original 10 RUCA categories into three, we divided census tracts within urbanized areas into two categories based on land area. This resulted in four community type categories along the rural-urban continuum: high density urban, lower density urban, suburban/small town, and rural [19, 25]. Veterans were assigned community types based on the census tracts associated with their addresses when they entered the VADR cohort. Only those addresses that we were able to successfully geocode were assigned community types and included in the subgroup analysis.

*Race/ethnicity:* We used self-identified individual race/ethnicity to assess variations in utilization outcomes trends across racial/ethnic groups. Four race/ethnicity categories with sufficient data were considered for comparison: Non-Hispanic White, Non-Hispanic Black, Hispanic, and Non-Hispanic Asian-American Pacific-Islander & Native-Indian American (AAPI). Those with missing race/ethnicity information were dropped from this subgroup analysis.

*Low income and disability:* We created a low income/disability variable as a proxy for socioeconomic status using VA established priority groups, based on veterans’ military service history, disability, income, and eligibility for Medicaid or other VA benefits [26]. The low income/disability variable, categorized as, disabled, low income but non-disabled, and neither, was used to characterize veterans with and without medication fills.

### Statistical analysis

We described the demographic characteristics of the veterans with diabetes in our analytic cohort. We used interrupted time series design [27], a quasi-experimental approach, to compare outcome trends at different phases of the pandemic. The interrupted time series design is an alternative to the randomized controlled designs, and leverages observational data and natural experiments in the event of an external shock, such as the COVID-19 pandemic, to assess impact of the shock. We visually assessed the assumptions of stationarity and lack of outliers, required by the design, by generating time series plots and visualizing the longitudinal trends.

Log-linear generalized additive regression model was used to compare the outcomes, rates of HbA1C tests, in-person visits (all outpatients and primary care only), telehealth visits (all outpatients and primary care only) and prescription fills (for diabetes and hypertension medications) with a binary variable indicating pre- and post-pandemic as the main exposure. Secular trends were modeled with a penalized cubic spline with the smoothing term selected using restricted maximum likelihood [28, 29]. To further ensure that there is no residual autocorrelation in the fitted model, we plotted the autocorrelation function of the residuals along with 95% confidence interval. In the subsequent analyses, we also fitted the regression models with a categorical exposure variable to indicate the three different phases in the first year of the pandemic, March–June 2020, July–December 2020, and January–March 2021, with pre-pandemic phase (March 2018–February 2020) as the reference category. Incidence rate ratios along with 95% confidence intervals were reported. Predicted monthly rates and 95% confidence intervals were also generated for each comparison phase.

#### Sub-group analyses

To assess whether disruptions in care due to COVID-19 had a differential impact among those with poor control compared to those with good control, we conducted analyses by stratifying the veterans based on their pre-pandemic glycemic control. To compare whether the associations differed by geography, we compared monthly rates of utilization outcomes stratified by community type among veterans with geocoded addresses. Finally, we also conducted the analyses stratified by self-identified race/ethnicity. All analyses were conducted using statistical software R (packages ‘mgcv’ and ‘ggeffects’). Statistical significance was two tailed at significance level of 0.05.

## Results

Table 1 shows the demographic characteristics of 733,006 veterans with well-documented diabetes. The mean age as of March 2018 was 67 years, with more than 50% over the age of 60. Veterans were pre-dominantly male (94%) and non-hispanic White (69%) followed by non-hispanic Black (21%) and 6% Hispanic. Among these veterans, 40% were categorized as low-income (not disabled), and 38% were disabled. Among 648,213 veterans who had pre-pandemic glycemic control data, 282,666 (43%) had HbA1c > 9.0, 224,658 (35%) had HbA1c between 6.5 and 8.9, 78,762 (12%) had HbA1c between 5.7 and 6.49, and 50,075 (7.7%) had HbA1c < 5.7. In this sample, 17.8% veterans were from rural communities, 24.7% from suburban, and 38.1% from urban communities, with 19% missing community type. Only 55% of the veterans in the cohort filled prescriptions for diabetes and 76% filled prescriptions for hypertension from VA pharmacy during March 2018–2021. Veterans with prescription fills were similar in sex and low-income status compared to those without (Table S3). However, those with prescription fills were slightly younger (76% over 60 years vs 83%) and predominantly non-hispanic Black (22% vs 16%).

**Table 1:** Summary characteristics of veterans from a large national cohort with well documented new type 2 diabetes prior to March 2018 and at least 1 primary care visit between March 2018 and March 2021

Baseline characteristics	Veterans with type 2 diabetes
	***n*** = 733,006
**Age at 2018, mean (sd) years**	67 (11.6)
**Age categories, n (%)**	
29–44	27,062 (3.7%)
45–59	137,984 (18.8%)
60–75	399,122 (54.5%)
75+	168,831 (23.0%)
**Gender, n (%)**	
Male	689,883 (94.0%)
Female	43,108 (6.0%)
**Race ethnicity, n(%)**	
Non-Hispanic White	475,737 (69.0%)
Non-Hispanic Black	146,608 (21.0%)
Hispanic	45,677 (6.6%)
Non-Hispanic Asian	6755 (0.9%)
Non-Hispanic Native Hawaiian or other Pacific	7517 (1.1%)
Non-Hispanic American Indian or Alaska Native	7135 (1.0%)
Missing	43,577 (5.9%)
**Pre-pandemic HbA1c control, n (%)**	
HbA1c < 5.7	50,075 (7.0%)
HbA1c (5.7–6.49)	78,762 (11.0%)
HbA1c 6.5–8.99	224,658 (31.0%)
HbA1c > 9	282,666 (39.0%)
Missing	84,793 (12.0%)
**Community type, n (%)**	
High density urban	69,129 (9.4%)
Lower density urban	210,273 (28.7%)
Suburban	130,995 (24.7%)
Rural	180,762 (17.8%)
Missing	141,847 (19.4%)
**Low income and disability flag, n(%)**	
Low income	288,725 (39.6%)
Disabled	276,090 (37.9%)
None of the above	163,350 (22.4%)
Missing	4841 (0.6%)

Since March 2020, there were sharp reductions in the monthly rates of HbA1C measurements and in-person outpatient visits, with the lowest rates in April 2020, Fig. 1. Telehealth visits increased starting March 2020, peaking in April 2020 and slowly declining throughout the first year of the pandemic as in-person visits returned. Most telehealth visits were via telephone rather than video. Among veterans who filled their prescriptions in VA pharmacies, we saw no major changes in fill rates after the pandemic onset for hypertension medication, although fill rates for diabetes medication showed a slight increase after July 2020. Trend plots (Figs. S1, S2 and S3) stratified by pre-pandemic glycemic control, race/ethnicity and community types showed similar patterns.

**Fig. 1:**
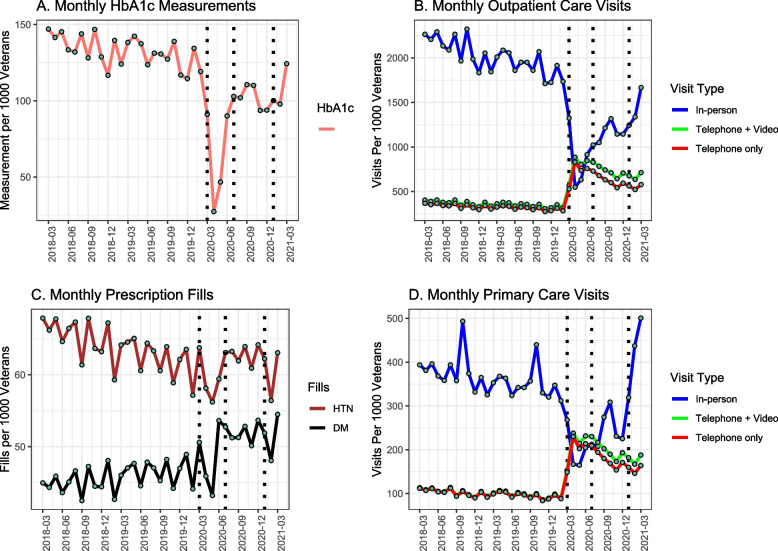
Trend plots visualizing monthly rates of healthcare utilization outcomes across pre-pandemic (March 2018 through February 2020) and post-pandemic (March 2020 through March 2021) periods. Vertical dotted lines indicate the onset of the early pandemic phase on March 2020, beginning of the mid phase on July 2020, and beginning of the late phase on January 2021. DM = Type 2 Diabetes, HTN = Hypertension

Rate ratios comparing monthly rates of utilization outcomes pre-pandemic (March 2018 through February 2020) and throughout the first year of the pandemic starting on March 2020 are reported in Table 2. We observed a 57% reduction in monthly rates of HbA1c measurements starting March 2020 compared to the pre-pandemic period. Reductions were greatest in the first 3 months, March–June 2020 with monthly predicted rate of 60 (95%CI,50–70) per 1000 veterans compared to 130 (95%CI,110–140) prior to March 2020. By June 2020, monthly rates of HbA1c measurements had largely returned to pre-pandemic rates (110;95%CI,90–130 per 1000 veterans). In the first year of the pandemic, in-person outpatient visits decreased by 56%, while telehealth visits increased by more than two-fold. Monthly rates of in-person visits decreased from 1830 visits (95%CI,1640–2040) to 810 (95%CI,710–930) per 1000 in March–June 2020 and to 1190 (95%CI,1040–1360) July–December 2020, before returning to pre-pandemic rates on January 2021. However, telehealth visits consistently increased throughout March 2020–2021 from 330 (95%CI,300–350) pre-pandemic to 770 (95%CI,720–820) visits per 1000. Overall combined in-person and telehealth outpatient visits decreased in the initial 3 months of the pandemic from 2130 (95%CI,2000–2280) to 1630 (95%CI,1520–1760) per 1000 and rebounded to pre-pandemic rates starting in July 2020. We observed similar trends when the visits were limited to primary care. Monthly rates of prescription fills for diabetes and for hypertension medications did not differ from pre-pandemic period. Plot of the autocorrelation functions (Fig. S6) suggested no evidence of residual autocorrelation, increasing our confidence in the model assumptions.

**Table 2:** Incidence rate ratios (RR)^+^ and 95% confidence interval comparing utilization outcomes after the onset of the pandemic on March 2020 through March 2021 to pre-pandemic period between March 2018 and February 2020

	March 2018–February 2020	March 2020 – March 2021	March 2018–February 2020	March 2020–June 2020	July–December 2020	January–March 2021
**HbA1C measurements**	Reference	0.43 [0.28, 0.65]	Reference	0.47 [0.36, 0.60]	0.86 [0.66, 1.12]	0.92 [0.65, 1.30]
**All outpatient care visits**		0.75 [0.66, 0.86]		0.77 [0.69, 0.85]	0.92 [0.81, 1.04]	1.04 [0.87, 1.24]
**In-person outpatient care visits**		0.44 [0.34, 0.56]		0.44 [0.37, 0.53]	0.65 [0.53, 0.79]	0.81 [0.62 1.05]
**Telehealth outpatient care visits**		2.35 [2.11, 2.62]		2.44 [2.17, 2.75]	2.26 [1.96, 2.60]	2.27 [1.94, 2.66]
**In-person primary care visits**		0.59 [0.44, 0.80]		0.59 [0.49, 0.71]	0.75 [0.62, 0.91]	1.32 [1.03, 1.68]
**Telehealth primary care visits**		2.35 [2.10, 2.62]		2.34 [2.08, 2.63]	2.32 [2.03, 2.65]	2.19 [1.83, 2.63]
**Diabetes prescription fills**		1.05 [0.99, 1.12]		1.02 [0.96, 1.09]	1.09 [1.02, 1.17]	1.07 [0.98, 1.17]
**Hypertension prescription fills**		1.01 [0.95, 1.07]		0.99 [0.94, 1.04]	0.99 [0.94, 1.04]	1.05 [0.99, 1.13]

^+^RRs were adjusted for non-linear secular trends

**Fig. 2:**
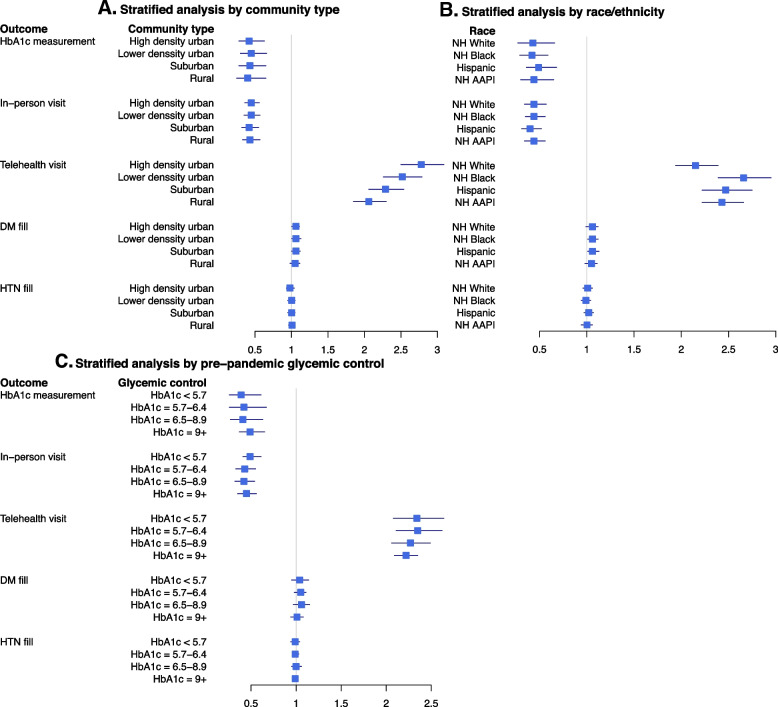
Sub-group analyses. Rate ratios comparing utilization outcomes after the onset of the COVID-19 pandemic (March 2020–2021) to those prior to the pandemic (March 2018–February 2020): (**A**.) stratified by community type, (**B**.) stratified by race and ethnicity, and (**C**.) stratified by pre-pandemic glycemic control. NH = non-Hispanic, AAPI = Asian American, Native American Indian, Pacific Islanders. DM = Diabetes, HTN = Hypertension

Stratified analyses (Fig. 2) showed differential impacts on telehealth visits by community type and race/ethnicity. Increase in monthly rates of telehealth visits starting March 2020 was lowest in rural community (RR = 2.06;95%CI,1.85–2.30) and highest in high density urban community (RR = 2.78;95%CI,2.50–3.09). Moreover, rates of telehealth visits increased most among non-hispanic Black veterans (RR = 2.66;95%CI,2.39–2.95) followed by Hispanics and non-Hispanic AAPI, and least among non-hispanic White (RR = 2.15;95%CI,1.94–2.38). However, we did not observe any difference in the associations by geography or race on HbA1c measurements, medication fills, and in-person visits. When stratified by pre-pandemic glycemic controls, relative rates of monthly HbA1c measurements were consistent across the strata. Similarly, in-person visits decreased and telehealth visits increased consistently for all levels of pre-pandemic glycemic control. There was also no evidence of variable changes in prescription fills.

## Discussion

In this large, national cohort of US veterans with diabetes diagnosed in the decade prior to the COVID-19 pandemic, we quantified the reduction in healthcare utilization after the onset of the pandemic compared to the pre-pandemic period. We observed fewer HbA1c measurements and fewer in-person outpatient visits in the first year of the pandemic period, with most pronounced reductions in March–June 2020, when nearly all elective procedures and routine care were reduced nationally due to public health protocols and pandemic demands on the healthcare system. Telehealth visits increased consistently starting March 2020, with important variation in uptake by different communities and racial groups. While there was substantial reduction in total number of outpatient visits March–June 2020, it returned to pre-pandemic levels starting July 2020. Trends in prescription fills for diabetes and hypertension medications, however, remained stable throughout the study period.

We observed disparate uptake of telehealth visits, with substantially lower uptake in rural communities compared to other community types, while urban communities had the largest increase. While we did not find disparate utilization for HbA1c measurements and prescription fills by race, we did see the highest increase in telehealth utilization among Black and lowest increase among White veterans. Our analyses further showed that utilization did not vary according to pre-pandemic glycemic levels, suggesting that the pandemic impacted veterans with diabetes across all levels of pre-pandemic control. Our findings are consistent with pior research that investigated utilizations in chronic diseases after external shock from a natural disaster [6, 30]. Since the onset of the pandemic, the overall trends of reduced in-person visits and increased telehealth visits among veterans who received medical care from VA health centers [2, 17] and among older veterans with diabetes [31] have been reported. However, our study is among the first to investigate variations in the impact of the pandemic on veterans with diabetes nationwide.

The trends in reduced utilization, including HbA1c testing and in-person visits, among veterans with diabetes are concerning as they highlight the missed opportunities to manage the chronic condition, which can result in worse outcomes including poor diabetes control [32–34]. While increased telehealth visits partially compensated for the missed in-person visits by July 2020, it is unclear whether replacement of in-person visits with telehealth helps in disease management, such as routine HbA1c measurement, among patients with diabetes. Furthermore, substantial geographic and racial variation in telehealth uptake is likely a premonition for future disparities in long-term clinical complications among this patient population.

While Black and Hispanic racial groups in urban communities have disproportionately experienced the burden of the pandemic [35], there is a mixed evidence indicating disparities on healthcare utilization through the pandemic [36, 37]. Our finding on lower telehealth utilization among rural residents is consistent with observed trends among patients with chronic diseases [38]. Limited broadband availability and low preparedness prior to the pandemic are potential key structural barriers to telehealth utilization among rural communities [39]. However, the increased telehealth utilization by Black veterans, who primarily resided in urban areas, is contrary to previous findings [40]. Further research is needed to understand whether telehealth could be helpful in mitigating health disparities.

While the pandemic disrupted routine healthcare utilization among veterans with diabetes, their access to medications were unaffected. Further, while in-person visits decreased since the pandemic onset, telehealth visits have increased. The rise in telehealth visits along with mail-order pharmacy could have played a role in ensuring that the medication orders were filled in timely manner with none to minimal disruption on the prescription fills.

### Limitations

Only 60% of the veterans with type 2 diabetes had filled their diabetes or hypertension medication from a VA pharmacy, limiting the generalizability of our findings. We used baseline addresses at the entry to VADR cohort, therefore we could not account for veterans who might have moved. While the strength of the analytical design is that we leveraged quasi-experimental technique within interrupted time series, there could be other plausible explanations of the observed associations. Finally, veterans are not representative of the general US population. Veterans who consistently use the VA may also have behaviors that prevented their healthcare utilization than those who do not. By limiting our analyses to VA EHR we may be missing utilization at non-VA sources of medical care.

## Conclusion

Healthcare utilization among veterans with diabetes was disrupted in the initial months of the COVID-19 pandemic before rebounding to pre-pandemic levels. There were large reductions in monthly rates of HbA1c measurements and in-person outpatient visits, whereas telehealth visits nearly doubled with substantial disparities by community type and race/ethnicity.

## Supplementary Information

**Additional file 1: Table S1.** Primary and secondary decision support identifiers (stop codes) for different delivery types. **Table S2.** Diabetes and hypertension medications, based on annual VA national formulary, considered for prescriptions fill analysis. **Table S3.** Summary characteristics of the analytic cohort among veterans from a large national cohort with well documented new type 2 diabetes prior to March 2018 and at least 1 primary care visit between March 2018 and March 2021, with and without medication fills. **Fig. S1.** Stratified trend plots comparing monthly rates of HbA1c measurements in the pre-pandemic period (March, 2018 - February, 2020) to those in the post-pandemic period (March, 2020 – March, 2021). Vertical dotted lines indicate the onset of the pandemic (phase 1) on March, 2020. NH = non-Hispanic, AAPI = Asian American, Native American Indian, Pacific Islanders. **Fig. S2.** Stratified trend plots comparing monthly rates of in-person outpatient visits in the pre-pandemic period (March, 2018 - February, 2020) to those in the post-pandemic period (March, 2020–2021). Vertical dotted lines indicate the onset of the pandemic (phase 1) on March, 2020. NH = non-Hispanic, AAPI = Asian American, Native American Indian, Pacific Islanders. **Fig. S3.** Stratified trend plots comparing monthly rates of telehealth outpatient visits in the pre-pandemic period (March, 2018 - February, 2020) to those in the post-pandemic period (March, 2020–2021). Vertical dotted lines indicate the onset of the pandemic (phase 1) on March, 2020. NH = non-Hispanic, AAPI = Asian American, Native American Indian, Pacific Islanders. **Fig. S4.** Stratified trend plots comparing monthly rates of diabetes prescription fills in the pre-pandemic period (March, 2018 - February, 2020) to those in the post-pandemic period (March, 2020–2021). Vertical dotted lines indicate the onset of the pandemic (phase 1) on March, 2020. NH = non-Hispanic, AAPI = Asian American, Native American Indian, Pacific Islanders. DM = Diabetes. **Fig. S5.** Stratified trend plots comparing monthly rates of hypertension prescription fills in the pre-pandemic period (March, 2018- February, 2020) to those in the post-pandemic period (March, 2020–2021). Vertical dotted lines indicate the onset of the pandemic (phase 1) on March, 2020. NH = non-Hispanic, AAPI = Asian American, Native American Indian, Pacific Islanders. HTN = Hypertension. **Fig. S6.** Autocorrelation function plots of the residuals from the models comparing outcomes in the pre-pandemic period (March, 2018 - February, 2020) to those in the post-pandemic period (March, 2020 –March, 2021). Dotted blue line represents 95% confidence interval assuming moving average process. ACF = Autocorrelation function.

## Data Availability

Data used in this study may be obtained from a third party and are not publicly available. Access to VA electronic health records data is limited to researchers with active, VA appointments and an IRB-approved protocol. Once a researcher has a VA appointment and IRB approval, the VA has a comprehensive infrastructure to support secure and remote access to data via the VINCI platform. Additionally, deidentified datasets from the VA Diabetes Risk cohort can be established and shared with appropriate IRB approval and data use agreements by contacting Dr. Lorna Thorpe (Lorna.Thorpe@nyulangone.org).

## Abbreviations

VA: Veterans administration
HbA1c: Glycohemoglobin
EHR: Electronic health records
VADR: VA Diabetes Risk
LEAD: Location, Environmental Attributes, and Disparities
CDC: Centers for Disease Control and Prevention
RUCA: Rural-Urban Commuting Area
AAPI: Asian-American Pacific-Islander & Native-Indian American
