# Supplementary material for: Disparities in routine healthcare utilization disruptions during COVID-19 pandemic among veterans with type 2 diabetes
Source: BMC Health Serv Res. 2023 Jan 16;23:41. doi: 10.1186/s12913-023-09057-8 (PMC9842402; doi:10.1186/s12913-023-09057-8)
Supplement: Supplementary file 1 — Additional file 1: Table S1. Primary and secondary decision support identifiers (stop codes) for different delivery types. Table S2. Diabetes and hypertension medications, based on annual VA national formulary, considered for prescriptions fill analysis. Table S3. Summary characteristics of the analytic cohort among veterans from a large national cohort with well documented new type 2 diabetes prior to March 2018 and at least 1 primary care visit between March 2018 and March 2021, with and without medication fills. Fig. S1. Stratified trend plots comparing monthly rates of HbA1c measurements in the pre-pandemic period (March, 2018 - February, 2020) to those in the post-pandemic period (March, 2020 – March, 2021). Vertical dotted lines indicate the onset of the pandemic (phase 1) on March, 2020. NH = non-Hispanic, AAPI = Asian American, Native American Indian, Pacific Islanders. Fig. S2. Stratified trend plots comparing monthly rates of in-person outpatient visits in the pre-pandemic period (March, 2018 - February, 2020) to those in the post-pandemic period (March, 2020–2021). Vertical dotted lines indicate the onset of the pandemic (phase 1) on March, 2020. NH = non-Hispanic, AAPI = Asian American, Native American Indian, Pacific Islanders. Fig. S3. Stratified trend plots comparing monthly rates of telehealth outpatient visits in the pre-pandemic period (March, 2018 - February, 2020) to those in the post-pandemic period (March, 2020–2021). Vertical dotted lines indicate the onset of the pandemic (phase 1) on March, 2020. NH = non-Hispanic, AAPI = Asian American, Native American Indian, Pacific Islanders. Fig. S4. Stratified trend plots comparing monthly rates of diabetes prescription fills in the pre-pandemic period (March, 2018 - February, 2020) to those in the post-pandemic period (March, 2020–2021). Vertical dotted lines indicate the onset of the pandemic (phase 1) on March, 2020. NH = non-Hispanic, AAPI = Asian American, Native American Indian, Pacific Islanders [file 12913_2023_9057_MOESM1_ESM.docx]

**Supplement for “Disparities in routine healthcare utilization disruptions during COVID-19 pandemic among veterans with type 2 diabetes”**

**Authors:**

Samrachana Adhikari, PhD^1^; Andrea R. Titus, PhD^1^; Aaron Baum, PhD^2^; Priscilla Lopez, PhD^1^; Rania Kanchi, MPH^1^; Stephanie L. Orstad, PhD^3^; Brian Elbel, PhD, MPH^1,4^; David C. Lee, MD^1,5^; Lorna E. Thorpe, PhD^1^; Mark D. Schwartz, MD^1,6^

**Affiliations:**

^1^Department of Population Health, New York University Grossman School of Medicine, New York, NY

^2^Department of Global Health, Icahn School of Medicine at Mount Sinai, New York, NY

^3^Department of Medicine, New York University Grossman School of Medicine, New York, NY

^4^Wagner Graduate School of Public Service, New York University, New York, NY

^5^Ronald O. Perelman Department of Emergency Medicine, NYU Grossman School of Medicine, New York, NY

^6^VA New York Harbor Healthcare System, New York, NY

**Corresponding author:**

Samrachana Adhikari, PhD

E-mail address: [samrachana.adhikari@nyulangone.org](mailto:samrachana.adhikari@nyulangone.org)

Telephone: 646-501-3647

Full postal address: 180 Madison Avenue, 4^th^ Floor, #4-54, New York, NY 10016

| **Table S1:** Primary and secondary decision support identifiers (stop codes) for different delivery types. | | | | | |  |  |
| --- | --- | --- | --- | --- | --- | --- | --- |
| **Clinic Type** | **Delivery type** | **Primary Stop Code Number** | | **Secondary Stop Code Number** | |  |  |
| **All outpatient care** | In-person* | - | | - | |  |  |
|  | Telephone | 103, 147, 148, 178, 181, 182, 199, 216, 221, 224, 229, 324, 325, 326, 338, 424, 425, 428, 527, 528, 530, 536, 537, 542, 545, 546, 579, 584, 597, 611, 686 | | **-** | |  |  |
|  |  |  |  |  |  |  |  |
|  |  |  |  |  |  |  |  |
|  | Video | - | | 136, 137, 179, 644, 645, 648, 690, 692, 693, 708 | |  |  |
|  |  |  |  |  |  |  |  |
|  | Secure message | - | | 719 | |  |  |
| **Primary Care** | In-person | 323 | | 185, 186, 323, 000 (blank) | |  |  |
|  | Telephone | 338 | | 117, 160, 185, 186, 323 | |  |  |
|  | VA video connect | 160, 323 | | 179 | |  |  |
|  | Clinical video telehealth at VA station | 301, 323 | | 692, 693 | |  |  |
|  | Secure message | 160, 186, 301, 323, 348 | | 719 | |  |  |
|  |  |  | |  | |  |  |
| * any outpatient encounters not categorized as telephone or video, is considered in-person visit for all outpatient care.   \| **Table S2:** Diabetes and hypertension medications, based on annual VA national formulary, considered for prescriptions fill analysis. \| \| \| --- \| --- \| \| **Diabetes medications** \| Chlorpropamide, Glipizide, Glyburide, Glimepiride, Metformin, Repaglinide, Nateglinide, Rosiglitazone, Pioglitazone, Sitagliptin, Saxagliptin, Linagliptin, Alogliptin, Canagliflozin, Dapagliflozin, Acarbose, Miglitol, Colesevelam, Insulin, Empagliflozin, Dextrose Squeeze Tube (Otc), Glucagon, Liraglutide (Victoza Only), Semaglutide \| \| **Hypertension medications** \| Acebutolol, Atenolol, Bisoprolol, Carvedilol, Esmolol, Labetalol, Metoprolol, Nadolol, Nebivolol, Penbutolol, Propranolol, Sotalol, Bumetanide, Chlorthalidone, Chlorothiazide, Ethacrynate, Furosemide, Hydrochlorothiazide, Indapamide, Methyclothiazide, Metolazone ,Torsemide, Benazepril, Captopril, Enalapril, Fosinopril, Lisinopril, Moexipril, Perindopril, Quinapril Hydrochloride, Ramipril, Trandolapril, Alfuzosin, Doxazosin Mesylate, Prazosin Hcl, Tamsulosin, Terazosin Hcl, Amlodipine, Diltiazem Hcl, Felodipine, Nicardipine, Nifedipine, Verapamil Hcl, Sacubitril/Valsartan, Clonidine, Hydralazine Hcl, Hydralazine/Isosorbide, Methyldopa, Methyldopate Hcl, Minoxidil, Reserpine, Sodium Nitroprusside, Candesartan, Irbesartan, Losartan, Telmisartan, Valsartan \| | | | | | |  |  |
| **Table S3:** Summary characteristics of the analytic cohort among veterans from a large national cohort with well documented new type 2 diabetes prior to March 2018 and at least 1 primary care visit between March 2018 and March 2021, with and without medication fills. | | | | | | | |
| **Baseline characteristics** | | | **Veterans with prescription fills** | | **Veterans without prescription fills** | | |
|  | | | **Overall**  **(n = 615,432)** | | **Overall**  **(n = 117,574)** | | |
| **Age at 2018, mean (sd) years** | | | 66.7 (11.3) | | 71.1 (12.6) | | |
| **Age categories, n (%)** | | |  | |  | | |
| 29 - 44 | | | 23212 (3.7%) | | 3850 (3.3%) | | |
| 45 - 59 | | | 122312 (19.8%) | | 15672 (13.3%) | | |
| 60 - 75 | | | 346545 (56.3%) | | 52577 (44%) | | |
| 75+ | | | 123356 (20.0%) | | 45475 (39%) | | |
| **Gender, n (%)** | | |  | |  | | |
| Male | | | 579662 (94.2%) | | 110221 (93.7%) | | |
| Female | | | 35757 (5.8%) | | 7351 (6.3%) | | |
| **Race ethnicity, n(%)** | | |  | |  | | |
| Non-Hispanic White | | | 395062 (67.9%) | | 80675 (74.4%) | | |
| Non-Hispanic Black | | | 129054 (22.2%) | | 17554 (16.2%) | | |
| Hispanic | | | 39246 (6.7%) | | 6431 (5.9%) | | |
| Non-Hispanic Asian | | | 5434 (0.9%) | | 1321 (1.2%) | | |
| Non-Hispanic Native Hawaiian or other Pacific Islander | | | 6398 (1.1%) | | 1119 (1.0%) | | |
| Non-Hispanic American Indian or Alaska Native | | | 5863 (1.0%) | | 1272 (1.1%) | | |
| Missing | | | 34375 (5.5%) | | 10523 (8.9%) | | |
| **Low income and disability flag, n(%)** | | |  | |  | | |
| Low income | | | 243052 (39.7%) | | 45673 (39.2%) | | |
| Disabled | | | 235786 (38.5%) | | 40304 (34.6%) | | |
| None of the above | | | 132894 (21.7%) | | 30456 (26.2%) | | |
| Missing | | | 3700 (0.6%) | | 1141 (0.9%) | | |

**Figure S1**: Stratified trend plots comparing monthly rates of HbA1c measurements in the pre-pandemic period (March, 2018- February, 2020) to those in the post-pandemic period (March, 2020 – March, 2021). Vertical dotted lines indicate the onset of the pandemic (phase 1) on March, 2020. NH = non-Hispanic, AAPI = Asian American, Native American Indian, Pacific Islanders.

**Figure S2:** Stratified trend plots comparing monthly rates of in-person outpatient visits in the pre-pandemic period (March, 2018- February, 2020) to those in the post-pandemic period (March, 2020 –March, 2021). Vertical dotted lines indicate the onset of the pandemic (phase 1) on March, 2020. NH = non-Hispanic, AAPI = Asian American, Native American Indian, Pacific Islanders.

**Figure S3**: Stratified trend plots comparing monthly rates of telehealth outpatient visits in the pre-pandemic period (March, 2018- February, 2020) to those in the post-pandemic period (March, 2020 – March, 2021). Vertical dotted lines indicate the onset of the pandemic (phase 1) on March, 2020. NH = non-Hispanic, AAPI = Asian American, Native American Indian, Pacific Islanders.

**Figure S4:** Stratified trend plots comparing monthly rates of diabetes prescription fills in the pre-pandemic period (March, 2018- February, 2020) to those in the post-pandemic period (March, 2020 – March, 2021). Vertical dotted lines indicate the onset of the pandemic (phase 1) on March, 2020. NH = non-Hispanic, AAPI = Asian American, Native American Indian, Pacific Islanders. DM = Diabetes.

**Figure S5:** Stratified trend plots comparing monthly rates of hypertension prescription fills in the pre-pandemic period (March, 2018- February, 2020) to those in the post-pandemic period (March, 2020 –March, 2021). Vertical dotted lines indicate the onset of the pandemic (phase 1) on March, 2020. NH = non-Hispanic, AAPI = Asian American, Native American Indian, Pacific Islanders. HTN = Hypertension.

**Figure S6**: Autocorrelation function plots of the residuals from the models comparing outcomes in the pre-pandemic period (March, 2018- February, 2020) to those in the post-pandemic period (March, 2020 –March, 2021). Blue line represents 95% confidence interval assuming moving average process. ACF: Autocorrelation function.
